# Freezing of gaze during action preparation under threat imminence

**DOI:** 10.1038/s41598-019-53683-4

**Published:** 2019-11-20

**Authors:** Lara Rösler, Matthias Gamer

**Affiliations:** Department of Psychology, Julius Maximilians University of Würzburg, Würzburg, Germany

**Keywords:** Stress and resilience, Visual system, Human behaviour

## Abstract

When confronted with threatening stimuli, animals typically respond with freezing behavior characterized by reduced movement and heart rate deceleration. Freezing-like responses during threat anticipation have also been observed in humans and are associated with anxiety. Recent evidence yet suggests that freezing does not necessarily reflect helpless immobility but can also aid the preparation of a threat escape. To investigate which further behavioral responses human freezing encompasses, we presented 50 young adults (10 male) with aversive stimuli that could sometimes be avoided while measuring gaze, cardiovascular and electrodermal activity. In trials in which the threat could be escaped, participants displayed reduced heart rate, increased electrodermal activity and reduced visual exploration. Furthermore, heart rate deceleration and restricted visual exploration predicted the speed of flight responses. These results provide evidence for freezing behavior in measures of visual exploration and suggest that such responding is adaptive in preparing the subsequent escape of approaching threats.

## Introduction

There is a fine line between appropriate avoidance behavior in response to threat and disproportionate avoidance characteristic of anxiety disorders. Rodents and non-human primates commonly respond with freezing as a reaction to relatively remote threat or threat-related cues^1–6^. This reduced mobility enhances chances of survival in life-endangering situations as immobile prey is less likely to be eaten than its moving peers^7,8^. Commonly characterized by both reduced body motion and heart rate deceleration, freezing does not always indicate defenseless immobility^9,10^. Freezing as compared to non-freezing rats show a heightened startle response, suggesting that freezing actively supports the detection of threat^11^ which, in turn, might improve fight-or-flight responses^12^. The animal literature hence seems to suggest that active freezing, preparing the animal for threat encounters, needs to be distinguished from tonic immobility, a temporary state of paralysis, which represents a passive last resort to an unavoidable threat^13^.

In humans, tonic immobility has been observed as an immediate response to traumatizing events and has therefore been suggested to be involved in the emergence of post-traumatic stress disorder (PTSD)^14,15^. Early-life stress has also been linked to increased freezing-like behavior in adolescents while reduced body sway in adults has been associated with a history of anxiety or trauma e.g.^16,17^. However, different experimental designs have recently demonstrated the benefits of postural freezing in humans as it potentially facilitates a subsequent threat avoidance^18–20^. Heart rate deceleration, a typical marker of freezing, is thus associated with better discrimination of low spatial frequency information^21^. The improved acuity of low spatial frequency vision occurs at the cost of high frequency recognition and might therefore facilitate fast but sometimes unnecessary escapes^21^. Given the accumulating evidence of a role of freezing that surpasses mere immobility, it has recently been suggested that freezing represents a necessary mechanism for action preparation^22^.

Research on the influence of threat on measures of visual exploration have primarily focused on the attentional capture of threatening stimuli. Accordingly, the use of dot-probe paradigms revealed shorter reaction times to threat-related or painful stimuli e.g.^23^ and difficulties disengaging from them^24,25^. This attentional bias towards threat has further been implicated in the maintenance of anxiety disorders (as reviewed in^26^). A meta-analysis on attentional biases towards pain confirmed that healthy participants and chronic pain patients show heightened attention towards impending pain signals^27^ suggesting a potential relationship between gaze patterns, commonly interpreted as markers of attention, and threat-related cues. In line with the idea that pain captures attention, painful stimulation was recently seen to trigger attentional shifts toward the simulation site^28^. However, as participants were not able to escape the painful shock, the study design did not allow for inferences about attentional processes during flight and freezing as a means to flight preparation.

Capitalizing on the link between threat and attentional capture, the aim of the current study was to investigate whether freezing responses can be discerned in measures of visual exploration and whether this freezing of gaze is contingent on the possibility to escape a threat. We therefore examined gaze patterns in trials in which a painful stimulus will certainly be administered and trials in which this threat can be actively avoided. To provide visual stimulation necessary to elicit visual exploration without biasing the observer’s attention towards a specific image region, we presented simplistic natural images to participants. The color of the fixation cross preceding the image onset indicated whether an unavoidable, an avoidable or no shock would be administered upon image disappearance. Throughout the entire experiment, we measured gaze, skin conductance and heart rate to investigate whether previously reported autonomic indicators of freezing would be accompanied by changes in visual exploration patterns. In line with the freezing for action hypothesis, we expected that freezing, as reflected in heart rate deceleration and a more focused visual exploration pattern characterized by a stronger central bias with fewer and longer fixations, would become most evident in flight trials when an action (i.e., an escape of the threat) is possible. We additionally postulated that a role of freezing in action preparation should manifest itself in faster reaction times in those trials in which freezing is most pronounced.

## Methods

### Participants

We invited 59 participants (11 males, age: *M* = 23.79 years, *SD* = 5.32 years) via a university-sponsored website targeted at participant recruitment from both the university and the local community to take part in our experiment. The study was conducted in line with the Declaration of Helsinki (version 2008) and approved by the Ethics committee of the Department of Psychology of the University of Würzburg. Each participant provided written informed consent prior to the experiment and received extra course credit or monetary compensation. All participants had normal or corrected-to-normal vision and reported no history of pain-related diseases. Because of problems with the experimentation sequences, 3 participants had to be excluded. Another 3 participants were excluded because of a large proportion of invalid eye-tracking trials (>30%) and 3 participants were excluded because they did not follow task instructions on the day of testing. The final sample thus consisted of 50 participants (see Table 1), who were characterized regarding general anxiety, anxiety sensitivity and depression using established self-report questionnaires (see experimental procedure for further details). With the current sample size, medium to large effects (as reported in^29^) in repeated measures analyses of variance (rmANOVAs, *f* = 0.25) could be detected with a power of 0.95 at a significance level of α = 0.05 when assuming a correlation of *r* = 0.50 between factor levels in the rmANOVA^30^ (see Supplemental Material). The experiment was not formally preregistered but de-identified data and analysis scripts can be found on the Open Science Framework (https://osf.io/n7vua/).

**Table 1:** Demographics of participants included in the final sample (*n* = 50).

	M	SD
Age (years)	23.37	5.16
Sex (male/female)	(10/40)	
Handedness (right/left/both)	(45/3/2)	
STAI	34.88	8.31
ASI	18.43	10.85
BDI	5.23	5.22

Note. STAI = State-Trait Anxiety Inventory, trait version, ASI = Anxiety Sensitivity Index, BDI = Beck-Depression-Inventory. Note that one participant did not complete the ASI and two participants failed to provide data for the BDI. The respective descriptive statistics are therefore based on 49 and 48 participants, respectively.

### Experimental paradigm and stimulation

**Figure 1:**
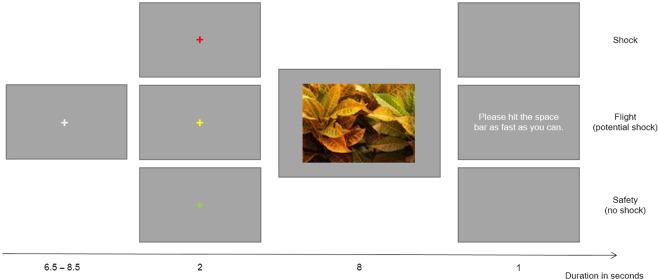
Illustration of the trial structure. Participants accomplished three different trial types (shock, flight and safety) that were announced by a color cue. Note: Size of fixation cross and color cue are not drawn to scale. Example image was taken from the McGill Calibrated Colour Image Database^31^.

Participants observed naturalistic images on a PC screen while their heart rate, skin conductance and eye movements were recorded. The stimulus set consisted of 60 naturalistic photographs (768 × 576 pixels, visual angle of 24.00° × 18.11° at the current viewing distance of 50 cm) which were taken from the McGill Calibrated Colour Image Database^31^. As we also examined attentional biases during threat anticipation, we used the same images as Schmidt and colleagues^28^ which were chosen for their uniform appearance (see Fig. 1 for an example). The experiment was programmed using Presentation® (Neurobehavioral Systems Inc., Version 18.1.) and presented on a grey background of a 24″ Asus VG248QE display (53.136 × 29.889 cm, 1920 × 1080 pixels, refresh rate 60 Hz). Pictures were presented pseudo-randomly, either in original or horizontally flipped orientation to account for effects of basic visual features on attentional orienting.

Visual stimulation consisted of an initially presented white fixation cross (6.5–8.5 s duration), a subsequent colored fixation cross (2 s duration), the naturalistic image (8 s duration) and a final grey screen which was either blank or contained a response prompt in the flight condition (1 s duration, see Fig. 1). The color of the second fixation cross indicated whether painful electrical stimulation would be either certainly (red; shock-condition), possibly (yellow; flight-condition) or certainly not (green; safety-condition) presented after stimulus offset. To simulate a possible escape to the threat, participants could avoid the shock by quickly hitting the space bar with their dominant hand upon stimulus offset in flight trials. During the first five of these flight trials, participants received a shock if they took longer than 240 ms to hit the space bar. In subsequent flight trials, the median reaction time of the first five flight trials was used as an individual threshold for each participant to ensure that flight attempts would sometimes but not always result in shocks^29^. Each trial type appeared 20 times in pseudo-randomized order, warranting that no more than three trials of the same type appeared after one another, resulting in a total of 60 trials.

Painful electrical stimulation was applied using a Digitimer DS7A constant current stimulator (Hertfordshire, United Kingdom) and a surface electrode attached to the dominant hand of the participant. Shock segments consisted of a train of three 2 ms square-wave pulses (separated by 50 ms) with alternating polarity. Shock intensity was individually adjusted before the experiment (for similar workup procedures see^32,33^). This involved repeatedly delivering electrical shocks starting at 0 mA and increasing in intensity in 0.1 mA increments. Each shock had to be rated verbally using a visual analog scale from 0 to 10 (VAS; 0 = “not painful at all” and 10 = “unbearably painful”). Participants were explicitly told that a VAS level of 4 should describe a clearly unpleasant yet bearable feeling. Whenever a shock was perceived as having an intensity of 4, the amperage was turned down to the minimum and the increasing electrical stimulation was rated anew. After three iterations, the mean amperage of the stimulations perceived as a VAS level 4 was calculated and, subsequently, an additional 50% of the respective amperage was added to determine the final stimulation used as a shock throughout the experiment. Across all participants, we applied an average amperage of 1.00 mA (*SD* = 1.14 mA).

### Experimental procedure

After signing the consent form, participants received task instructions and learned which fixation cross color was associated with which trial type. Subsequently, we attached heart rate and skin conductance electrodes to the chest and the non-dominant hand of participants respectively. The surface electrode for delivering electrical stimulation was attached to the dominant hand. Participants were then seated in a dimly lit sound-proof cabin and instructed to rest their heads on a chin rest to ensure minimal movement during the subsequent eye-tracking recordings. After adjusting the shock intensity, the eye-tracker was calibrated and the calibration was validated using a nine-point grid. The experiment was then started and participants completed all 60 trials.

### Questionnaires

After the experimental task, participants completed the German versions of the following self-report questionnaires: (1) The Beck-Depression-Inventory (BDI)^34^ which consists of 21 multiple-choice questions that assess symptoms of depression. Each question contains four possible answers ranging in intensity. To score the BDI, every answer is assigned a value between 0 (no indication of depression) to 3 (severe depression) and values are subsequently summed up. BDI sum scores thus range from 0 to 63. Two participants did not complete the BDI. Cronbach’s α as a measure of internal consistency was good in the current sample (α = 0.82). (2) Participants additionally completed the State-Trait Anxiety Inventory (STAI)^35^ in its trait version that allows for an assessment of trait anxiety. It consists of 20 short descriptions of emotional states which are supposed to be rated on a 4-point Likert scale from 1 (almost never) to 4 (almost always) depending on their regular occurrence. STAI sum scores range from 20 to 80 with higher levels indicating higher levels of trait anxiety. Cronbach’s α was excellent in the current sample (α = 0.90). (3) Finally, the Anxiety sensitivity index (ASI)^36^ was used to measure one’s fear of experiencing anxiety. Participants indicate the extent to which each of 16 anxiety-related statements reflect their personal experience on a 5-point Likert scale from 0 (very little) to 4 (very much). Sum scores therefore range from 0 to 64. One participant did not complete the questionnaire but for the remaining sample internal consistence was excellent (α = 0.91).

### Data recording and analysis

#### Heart rate

Heart rate (HR) was measured using a BIOPAC MP160 device (BIOPAC Systems, Inc., Goleta, CA, USA) with a sampling rate of 500 Hz. Electrocardiogram (ECG) electrodes were placed on the manubrium sterni and the left lower rib cage. The reference electrode was placed at the right lower rib cage. In order to derive heart rate from the ECG signal, we relied on previously established procedures^37,38^. In detail, the data were first filtered with a 2 Hz high-pass filter to remove slow signal drifts. R-Waves were then detected from the ECG data using a semiautomatic method and R-R-intervals were converted to HR (in beats per minute). Then, a second-by-second sampling was applied^39^ for an interval of −1 to 15 s relative to the color cue preceding image onset. The HR in the last second prior to cue onset served as baseline and the mean heart rate change was derived by subtracting the baseline from the HR score of each postcue second.

#### Electrodermal activity

Electrodermal activity (EDA) was measured with the same BIOPAC device at the thenar and hypothenar eminences of the participants’ non-dominant hand by a constant voltage system (0.5 V) using a bipolar recording with two Hellige Ag/AgCl electrodes filled with 0.05 M NaCl electrolyte. In order to allow for similar analyses as for the heart rate data, EDA data were downsampled to 20 Hz and averaged in bins of 1 s between −1 and 15 s relative to the color cue preceding image onset. The mean skin conductance in the last second prior to cue onset served as baseline and was subtracted from the values of each postcue second.

#### Eye-tracking

We tracked the participants’ right eye using a mounted EyeLink 1000 Plus system with a sampling rate of 1,000 Hz (SR Research Ltd., Ottawa, Canada). Eye-tracking data were initially processed using EyeLink’s standard parser configuration which defined saccades as eye movements exceeding a velocity threshold of 30°/s or an acceleration threshold of 8,000°/s^2^. Time periods with stable gaze between saccades were defined as fixations. To warrant baseline stability in all trials entering the analysis, we considered the last 300 ms before naturalistic scene onset as fixation baseline. We subsequently examined baseline stability for each participant by conducting an iterative outlier removal procedure separately for x- and y-baseline coordinates (for a similar procedure see^40–42^). Specifically, the smallest and the largest values were temporarily removed from the distribution and if one or both of these values were more than three standard deviations away from the mean baseline position of the remaining data, they were permanently excluded from the analysis. This procedure was repeated until no further exclusion had to be performed. Fixation x and y coordinates were then corrected for gaze drift by subtracting the baseline from the actual x and y coordinate values. Trials with an invalid baseline (6.9% of all trials) were excluded from all subsequent analyses of physiological responses and visual exploration behavior.

We looked at three different metrics to evaluate the dynamics of visual exploration during threat anticipation. To account for and investigate potential changes in gaze throughout the trial, we separated fixations in eight bins of 1 s each as a first step. We then calculated average fixation durations, number of fixations and average distance of all fixations from the screen’s center (in pixels) within these bins to investigate whether freezing behavior could be observed in eye movements by proxy of fewer and longer fixations which occurred closer to the center.

To illustrate the spatial pattern of fixations during picture viewing, we also calculated fixations density maps as a function of trial type. This was accomplished by calculating individual fixation density maps per trial which were smoothed with a Gaussian kernel of 1° of visual angle. These maps were normalized to a range of 0 to 1 and subsequently averaged within individuals before calculating average maps across all participants.

#### Statistical analysis

All autonomic and ocular measures were statistically evaluated using a repeated measures analyses of variance (rmANOVA). These analyses included the within-subject factors trial-type (flight, shock or safety) and bin (15 levels for HR and EDA and 8 levels for measures of visual exploration). Degrees of freedom were adjusted according to the Greenhouse-Geisser method to compensate for potential violations of the sphericity assumption. Effect sizes are reported as generalized η^2^ ^43^. Post-hoc tests were carried out to specifically compare the responses in flight trials to the other two conditions. Therefore, we calculated *t*-tests for each time bin and adjusted the *p*-values for multiple comparisons using the false discovery rate (FDR, Benjamini & Hochberg^44^).

In order to exploratively examine the influence of physiological and eye-tracking indices of freezing on behavioral responses, we calculated a generalized linear mixed model (GLMM) on response times in flight trials using the lme4 package (version 1.1–15)^45^ for R. Trials where participants did not respond within 1 s were excluded from the analysis (3.7%). Based on the results of the rmANOVAs (see below) as well as previous research using a comparable experimental paradigm^29^, we concentrated on the responses in the second half of the stimulus presentation, where differences in freezing between conditions were strongest. Thus, we averaged time bins 5 to 8 for skin conductance, heart rate, center bias, fixation duration and fixation number and included these variables as predictors per trial in the GLMM. Participant ID was included as random intercept to warrant a within-subject evaluation of predictors and to account for a general variability of response speed between participants. Model estimates were chosen to optimize the restricted maximum likelihood (REML) criterion and *p*-values for each predictor were obtained based on Satterthwaite’s approximation of degrees of freedom using the lmerTest package (version 2.0–25)^46^.

Finally, we investigated whether individual differences in anxiety levels were associated with freezing behavior during flight trials. To this end, we calculated correlation coefficients for the relationship between the ASI and STAI questionnaires and the different freezing measures. Similar to the GLMM approach above, we focused these analyses on the second half of the trial when anticipation was supposedly strongest. Thus, we calculated the average of the last four seconds of heart rate, skin conductance, fixation number, fixation duration and distance from center before stimulus offset and correlated these values with STAI and ASI sum scores. All data preprocessing and analyses were carried out in R (Version 3.3.3, Core Team, 2018) on an α level of 0.05.

## Results

### Physiological responses

**Figure 2:**
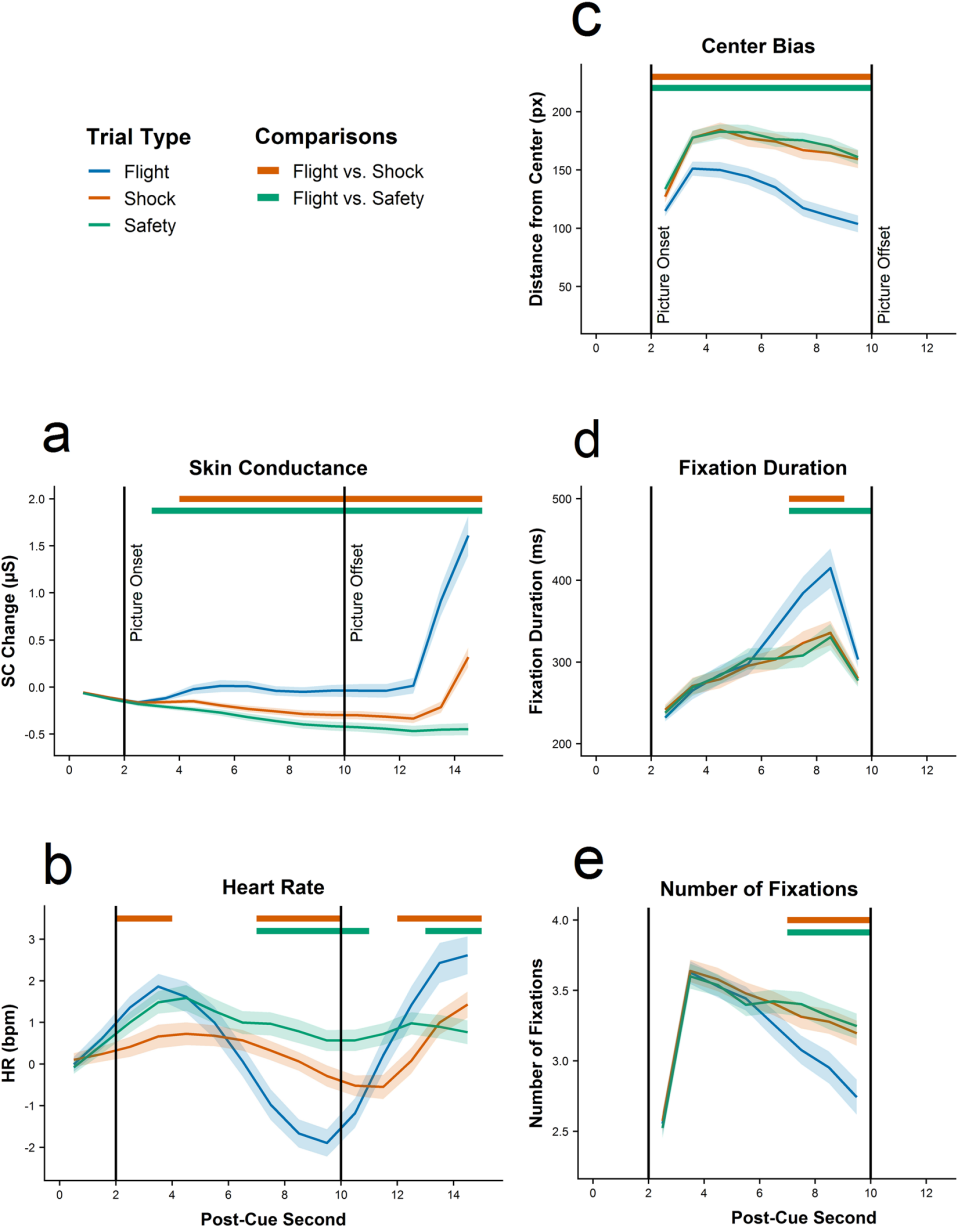
Mean changes in skin conductance level (**a**) and heart rate (**b**) relative to the last second prior to cue onset and average distance from center (**c**), fixation durations (**d**) and number of fixations (**e**) during picture presentation. Shaded areas denote standard errors of the mean. Time points with significant differences between the flight condition and the other two conditions (after false discovery rate correction) are displayed at the top of each figure as colored lines.

To assess whether physiological responses differed across trial types and over time, we performed a repeated-measures analysis of variance (rmANOVA) evaluating the influence of time bin (15 bins à 1 second) and trial type (shock vs. flight vs. safety) on skin conductance changes. A main effect of bin (*F*_(14,686)_ = 40.46, ε = 0.12, *p* < 0.001, η^2^ = 0.162), a main effect of trial type (*F*_(2,98)_ = 34.02, ε = 0.71, *p* < 0.001, η^2^ = 0.159) and a significant interaction between bin and trial type (*F*_(28,1372)_ = 38.72, ε = 0.08, *p* < 0.001, η^2^ = 0.215) suggest that temporal changes in electrodermal activity were modulated by trial type. Similarly, the rmANOVA on heart rate changes revealed a significant main effect of bin (*F*_(14,686)_ = 22.69, ε = 0.28, *p* < 0.001, η^2^ = 0.095) and a significant interaction between bin and trial type (*F*_(28,1372)_ = 14.06, ε = 0.22, *p* < 0.001, η^2^ = 0.070). There was, however, no statistically significant main effect of trial type (*F*_(2,98)_ = 1.61, ε = 0.91, *p* = 0.205, η^2^ = 0.011) suggesting that trial types do not per se elicit different cardiovascular responses but differ in the progress of these responses over time. Post-hoc tests, as depicted by colored lines above the graphs in Fig. 2, indicate that flight trials were associated with a marked increase in skin conductance that started shortly after picture onset and was maintained until (potential) shock delivery (see Table S1 for statistics of post-hoc tests). By contrast, a pronounced heart rate deceleration in flight trials only occurred during the second half of the picture viewing period and turned into an acceleration after picture offset.

### Visual exploration

**Figure 3:**
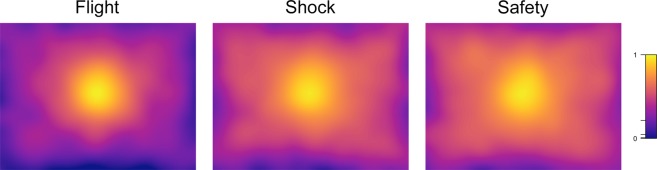
Smoothed (Gaussian kernel with *SD* = 1° of visual angle) fixation density maps illustrating the distribution of all fixations as a function of trial type (flight, shock, safety). Fixation density maps are displayed on a logarithmic scale.

Measures of visual exploration were analyzed in a similar fashion as the physiological data but since these measures can only be meaningfully interpreted during the picture viewing period, these analyses were restricted to eight time bins. The rmANOVA on the average distance of fixations from the screen’s center yielded a significant main effect of bin (*F*_(7,343)_ = 39.04, ε = 0.39, *p* < 0.001, η^2^ = 0.098), a significant main effect of trial type (*F*_(2,98)_ = 82.06, ε = 0.77, *p* < 0.001, η^2^ = 0.143) and a significant interaction of bin and trial type (*F*_(14,686)_ = 12.90, ε = 0.58, *p* < 0.001, η^2^ = 0.021). Similarly, we found a significant main effect of bin (*F*_(7,343)_ = 37.60, ε = 0.33, *p* < 0.001, η^2^ = 0.137), a significant main effect of trial type (*F*_(2,98)_ = 6.04, ε = 0.74, *p* = 0.003, η^2^ = 0.016) and a significant main interaction of bin and trial type (*F*_(14,686)_ = 10.15, ε = 0.45, *p* < 0.001, η^2^ = 0.028) for fixation durations. We further detected a significant main effect of bin (*F*_(7,343)_ = 96.40, ε = 0.35, *p* < 0.001, η^2^ = 0.205), a significant main effect of trial type (*F*_(2,98)_ = 9.25, ε = 0.74, *p* < 0.001, η^2^ = 0.014) and a significant main interaction of bin and trial type (*F*_(14,686)_ = 10.28, ε = 0.53, *p* < 0.001, η^2^ = 0.019) for the number of fixations made. Figure 2, which displays significant differences between conditions as colored lines above the graphs, indicates a stronger center bias in flight trials throughout the entire viewing period (also see Table S1). By contrast, fixation durations and numbers in flight trials only differed from the respective values in shock and safety trials at the end of the picture viewing period. The observed increase in fixation durations and the related decrease in fixation numbers indicate a general reduction in visual exploration in anticipation of the flight response. This pattern is also evident in fixations density maps for all fixations as a function of trial type (flight, shock, safety, see Fig. 3).

### Prediction of flight behavior

Participants’ mean response time in flight trials was 298.45 ms (*SD* = 71.44 ms). A generalized linear mixed model (GLMM) revealed heart rate responses as well as center bias to be significantly related to response times in individual flight trials (Table 2). In detail, stronger heart rate decelerations as well as an increased center bias preceding picture offset (i.e., a reduced distance of fixations to the screen’s center) predicted faster responses. The other predictors were not significantly related to response times in a combined model, which might be partly due to intercorrelations between eye-tracking measures (see Table S2 in Supplementary Material).

**Table 2:** Parameters of a generalized linear mixed model predicting response times in flight trials.

Predictor	β	*SE*	*t*	*df*	*p*
(Intercept)	265.958	57.210	4.65	660.2	<0.001
Skin conductance	−0.410	1.160	−0.35	652.5	0.724
Heart rate	0.500	0.208	2.40	656.6	0.017
Center bias	0.084	0.022	3.83	615.1	<0.001
Fixation duration	−0.005	0.016	−0.34	662.5	0.736
Fixation number	1.059	3.002	0.35	663.6	0.724

Note. *p*-values for each predictor were obtained based on Satterthwaite’s approximation of degrees of freedom.

### Correlational analyses of anxiety and freezing behavior

Overall, correlations between anxiety and freezing behavior were small and did not reach statistical significance (see Table S3 in Supplementary Material). The only exception was a significant relationship of fixation numbers and ASI (*r* = −0.35, *p* = 0.015). However, when applying a Bonferroni procedure, which seems appropriate given the exploratory nature of the current analyses, this correlation is no longer statistically significant.

## Discussion

The current study investigated autonomic and gaze responses in the anticipation of a painful shock that was either unavoidable or could be escaped via a button press. We observed fewer and longer fixations, which were also closer to the center of the screen, in trials in which a threat escape was possible. This freezing response at the level of visual exploration was accompanied by a stark heart rate deceleration and an overall increase of electrodermal activity. Additionally, reductions in heart rate and visual exploration of the stimulus predicted the speed of the escape response. Our results therefore suggest that the observed freezing behavior in humans might play an important role in action preparation.

In trials in which an escape of the threat was possible, participants exhibited clearly distinct gaze patterns which consisted of a reduced average distance to the center of the screen and fewer but longer fixations. These freezing-like eye movements, that were demonstrated for the first time in the current study, possibly aid a faster escape of the subsequent threat. Indeed, a GLMM analysis revealed that heart rate deceleration and reduced visual exploration of the presented stimuli were significant predictors of flight reaction times. It remains to be tested, however, whether there is a causal link between reduced response times and visual freezing and by which means this possible causation is accomplished. A recent study suggested a relationship between non-visual freezing measures and improved perception, as they observed an association between decreases in heart rate and better discrimination of low spatial frequency stimuli^21^, potentially enabling crude but faster flight responses to threatening stimuli. Similarly, the expectation of pain at a specific location heightened multisensory processing as reflected by faster target detection at the pain-associated site^47,48^. Again, reduced scanning of the environment might have served as a preparatory mechanism enabling a subsequent harm prevention. Future studies should investigate whether a relationship between diminished visual exploration and faster reaction times also persists when the presented visual stimuli are more complex and realistic, for instance when displaying dynamic videos or being immersed in a virtual reality environment. Furthermore, it remains to be tested whether the currently observed potential advantage of reduced visual exploration persists when the environment has to be visually sampled in order to prepare appropriate flight responses.

The opportunity to actively escape the painful stimulation also led to clearly recognizable changes in the physiological reactions of our participants. We observed highest skin conductance levels in trials in which the electric shock could be avoided. This observation is in line with previous studies reporting higher electrodermal activity during threat anticipation^21,29,49^. Elevated skin conductance is a marker of sympathetic arousal and the observed increase can therefore likely be understood as increased arousal induced by the anticipation of threat. However, rather than the expectation of a painful stimulation per se, the preparation of a button press could have also triggered increased arousal levels. To elucidate whether and to what extent motor action planning contributes to sympathetic arousal, a safe button press condition in which no shock follows needs to be included in the experimental design.

Previously, Löw and colleagues^29^ had shown that attentive freezing, as indicated by fear bradycardia, exclusively took place when the threat could not be avoided. In contrast, we observed the most prominent heart rate deceleration when the painful stimulation following picture offset could be escaped via a button press. Our finding is supported by a study of Gladwin and colleagues^19^ which showed that fear bradycardia was strongly related to the preparatory state but only when a virtual gunshot resulting in painful electrical stimulation could be avoided. Which factors can explain these differential findings regarding the role of attentive freezing? The most striking difference between our experimental design and the study of Löw and colleagues is that our anticipatory state did not contain changing, dynamic stimuli but instead consisted of an 8 second viewing period of a static image. To observe changes with regard to threat proximity, Löw and colleagues used stimuli that increased in size to inform participants about threat imminence. One possible interpretation is that the dynamically approaching stimuli led to differential autonomic responses potentially by precisely informing participants about shock proximity. A closer examination of Löw and colleagues’ data yet shows that the active avoidance of threat was indeed associated with heart rate deceleration but not significantly more than the active button press in safe trials. This, in turn, suggests that the anticipation of a required button press, independent of whether a shock will follow or not, involves heart rate deceleration as a preparation for action. In the current study, we decided not to include a safe condition requiring fast responses since adaptive flight behavior is associated with the presence of threat. We can therefore not compare the anticipation of a button press in safe and unsafe trials. Overall, however, the strong fear bradycardia observed in flight trials which also predicted the speed of flight responses supports the action preparation function of freezing.

At first glance the notion of freezing as an adaptive preparatory flight mechanism is at odds with various animal and human studies which previously pointed to a relationship between anxiety and increased freezing behavior. Accordingly, rodents^50,51^ and non-human primates^6^ with high trait anxiety displayed more freezing behavior than their less anxious peers. Similarly, humans who reported higher state anxiety responded with more pronounced reductions in heart rate and body sway to angry versus neutral or happy faces^17^ and reduced postural sway when presented with a neutral target^52^. This increased freezing response is not limited to humans with comparatively high state anxiety but extends to patients with a history of panic disorder^53^ and aversive life events^54^. None of these experimental designs, however, allowed participants to escape the threatening situation. When we think of these freezing responses in terms of tonic immobility – a resigned acceptance of the approaching unavoidable threat – it becomes clear that these associations might point to an entirely different phenomenon. While highly anxious individuals might exhibit increased freezing as a sign of helplessness or resignation, freezing for action preparation, as measured in our study, might not be at all related to anxiety. Consequently, we failed to detect any relationship between anxiety measures and freezing behavior in the current sample. Similarly, Gladwin and colleagues who also investigated human freezing responses when an escape of the threat was possible failed to observe significant correlations between anxiety and freezing behavior^19^. The present results therefore emphasize the necessity to distinguish between different types of freezing responses: (1) tonic immobility which is linked to various anxiety disorders (e.g. social anxiety disorder^17^ and panic disorder^53^) and (2) freezing as a preparatory flight mechanism which is an adaptive response to a threat encounter.

The current investigation of cardiovascular, electrodermal and gaze responses to the anticipation of an electric shock that was either avoidable or unavoidable revealed that participants display increased freezing behavior when given the chance to escape the threat. This heightened freezing response was reflected in a stark heart rate deceleration, a simultaneous increase of electrodermal activity and reduced visual exploration of the presented stimuli. While our results thus provide evidence for freezing-like behavior in measures of visual exploration, the exact conditions under which this freezing of gaze occurs and the function that it serves need to be further examined. However, as we observed a correlation between heart rate deceleration and reduced scanning of the presented stimuli with the speed of the flight response, freezing might constitute an adaptive mechanism which serves as preparation for an adaptive response to imminent threat.

## Supplementary information

Supplementary Material
