# Supplementary material for: Freezing of gaze during action preparation under threat imminence
Source: Sci Rep. 2019 Nov 20;9:17215. doi: 10.1038/s41598-019-53683-4 (PMC6868270; doi:10.1038/s41598-019-53683-4)
Supplement: Supplementary file 1 — Supplementary Material [file 41598_2019_53683_MOESM1_ESM.docx]

**Freezing of gaze during action preparation under threat imminence**

Lara Rösler & Matthias Gamer

**Supplemental Material**

**Supplemental Methods**

*Power calculation*

Sample size calculations for the current study were based on previous studies using similar experimental paradigms (*n* = 27; Löw, Weymar, & Hamm, 2015) or relying on similar measures (*n* = 28; Schmidt, Gamer, Forkmann, & Bingel, 2018). On this basis, we decided to acquire valid data from 50 participants. Since a priori calculations of statistical power are difficult for experimental designs using multiple within-subject factors, we used the following strategy to do a post-hoc estimation of statistical power for the current study. Since we were mainly interested in revealing differences in the time course of physiological (skin conductance and heart rate) and eye-tracking measures (central bias, number and duration of fixations) as a function of trial type (flight, shock or safety), we reduced the two-factorial design to a one-factorial within-subjects design assuming the calculation of pairwise response differences (e.g., flight vs. shock or flight vs. safety) within each time bin. With the current sample size, such design permits to reveal medium effects (*f* = 0.25) at an alpha level of .05 with a statistical power greater than .95 when assuming a correlation of *r* = .50 between factor levels. This holds true for analyses with 15 time bins (physiological measures) as well as those with 8 bins (measures of visual exploration) even when applying nonsphericity correction across a large range of values (.35 < ε < 1.00).

**Table S1.** False discovery rate corrected post-hoc tests.

| Time in seconds |  | Skin Conductance | | Heart Rate | | Central Bias | | Fixation Duration | | Number of Fixations | |
| --- | --- | --- | --- | --- | --- | --- | --- | --- | --- | --- | --- |
|  |  | Flight vs Shock | Flight vs Safety | Flight vs Shock | Flight vs Safety | Flight vs Shock | Flight vs Safety | Flight vs Shock | Flight vs Safety | Flight vs Shock | Flight vs Safety |
| 0-1 | *t*  *p* | -0.208  .874 | 0.505  .616 | -0.510  .612 | 0.387  .750 | - | - | - | - | - | - |
| 1-2 | *t*  *p* | -0.372  .821 | 0.773  .475 | 1.336  .234 | 0.553  .672 | - | - | - | - | - | - |
| 2-3 | *t*  *p* | 0.159  .874 | 0.826  .475 | 2.485  .031 | 1.172  .463 | -3.668  .001 | -6.525  .000 | -1.754  .137 | -0.891  .603 | -0.332  .787 | 0.583  .711 |
| 3-4 | *t*  *p* | 1.621  .139 | 3.251  .003 | 2.843  .024 | 1.074  .480 | -6.102  .000 | -6.25  .000 | -0.687  .626 | -0.433  .762 | -0.272  .787 | 0.496  .711 |
| 4-5 | *t*  *p* | 2.769  .011 | 4.001  .000 | 1.974  .090 | 0.048  .962 | -6.753  .000 | -6.482  .000 | 0.606  .626 | 0.132  .896 | -0.923  .577 | -0.173  .863 |
| 5-6 | *t*  *p* | 3.376  .002 | 4.179  .000 | 0.715  .512 | -0.566  .672 | -6.086  .000 | -7.275  .000 | 0.147  .883 | -0.462  .762 | -0.574  .758 | 0.605  .711 |
| 6-7 | *t*  *p* | 4.043  .001 | 4.758  .000 | -1.187  .278 | -2.080  .092 | -6.450  .000 | -6.777  .000 | 2.377  .051 | 2.128  .077 | -2.070  .087 | -2.139  .075 |
| 7-8 | *t*  *p* | 4.005  .001 | 4.922  .000 | -3.283  .009 | -4.235  .001 | -7.750  .000 | -9.873  .000 | 3.809  .002 | 4.328  .000 | -3.446  .003 | -4.203  .000 |
| 8-9 | *t*  *p* | 4.037  .001 | 4.936  .000 | -4.409  .001 | -5.350  .000 | -9.473  .000 | -10.232  .000 | 4.431  .000 | 4.533  .000 | -4.114  .001 | -4.402  .000 |
| 9-10 | *t*  *p* | 3.836  .001 | 4.601  .000 | -3.795  .003 | -5.522  .000 | -10.250  .000 | -10.324  .000 | 2.301  .051 | 2.513  .041 | -5.479  .000 | -5.501  .000 |
| 10-11 | *t*  *p* | 3.708  .001 | 4.198  .000 | -1.550  .174 | -3.754  .002 | - | - | - | - | - | - |
| 11-12 | *t*  *p* | 3.658  .001 | 4.051  .000 | 1.694  .145 | -0.996  .487 | - | - | - | - | - | - |
| 12-13 | *t*  *p* | 3.973  .001 | 4.217  .000 | 2.613  .030 | 0.782  .597 | - | - | - | - | - | - |
| 13-14 | *t*  *p* | 6.837  .000 | 6.874  .000 | 2.707  .028 | 2.668  .026 | - | - | - | - | - | - |
| 14-15 | *t*  *p* | 6.745  .000 | 8.169  .000 | 2.477  .031 | 3.52  .003 | - | - | - | - | - | - |

**Table S2.** Pairwise correlations of fixed effects in the generalized linear mixed model predicting response times in flight trials.

|  | Skin Conductance | Heart  Rate | Center  Bias | Fixation  Duration |
| --- | --- | --- | --- | --- |
| Heart rate | -.012 |  |  |  |
| Center bias | .037 | -.026 |  |  |
| Fixation duration | .032 | .033 | .007 |  |
| Fixation number | .021 | .028 | -.256 | .799 |

*Note*. Measures were averaged across all time bins during the second half of the stimulus presentation (bin 5 to 8).

**Table S3.** Correlation coefficients between the self-report anxiety measures and freezing behavior.

|  | Center Bias | Fixation Duration | Fixation Number | Skin Conductance | Heart Rate |
| --- | --- | --- | --- | --- | --- |
| ASI | -0.239 | 0.279 | -0.347 | -0.033 | 0.089 |
| STAI | -0.197 | 0.054 | -0.085 | -0.097 | 0.055 |

*Note*. ASI = Anxiety Sensitivity Index; STAI = State-Trait Anxiety Inventory. Freezing measures were averaged across all time bins during the second half of the stimulus presentation (bin 5 to 8).
